# Suppressive effect of vitamin K_2_ (menatetrenone) against bone mineral density loss after radiotherapy in uterine cancer patients

**DOI:** 10.1007/s11604-025-01733-5

**Published:** 2025-01-24

**Authors:** Yuko Kaneyasu, Hisaya Fujiwara, Tomoyuki Akita, Junko Tanaka, Yuuka Shibata, Tomio Nakagawa, Iemasa Koh, Eiji Hirata, Maki Hyodo, Tadashi Miyamoto, Yuji Murakami, Ikuno Nishibuchi, Nobuki Imano, Yasushi Nagata, Yoshiki Kudo

**Affiliations:** 1 Department of Radiation Oncology, National Hospital Organization Fukuyama Medical Center, 4-14-17 Okinogami-Cho, Fukuyama, Hiroshima, 720-8520 Japan; 2 Department of Radiation Oncology, Graduate School of Biomedical Sciences, Hiroshima University, Hiroshima, Japan; 3 Department of Obstetrics and Gynecology, Chugoku Rosai Hospital, Hiroshima, Japan; 4 Department of Epidemiology, Infectious Disease Control and Prevention, Graduate School of Biomedical and Health Sciences, Hiroshima University, Hiroshima, Japan; 5 Department of Pharmaceutical Services, Hiroshima University Hospital, Hiroshima, Japan; 6 Department of Obstetrics and Gynecology, Graduate School of Biomedical Sciences, Hiroshima University, Hiroshima, Japan; 7 Hirata Ladies Clinic, Hiroshima, Japan; 8 Mother and Fetus Maki Clinic, Hiroshima, Japan; 9 Department of Orthopedic Surgery, National Hospital Organization Fukuyama Medical Center, Hiroshima, Japan; 10 Department of Radiation Oncology, Chugoku Rosai Hospital, Hiroshima, Japan

**Keywords:** Uterine cancer, Radiotherapy, Bone mineral density, Vitamin K_2_

## Abstract

**Purpose:**

This study aimed to investigate whether vitamin K_2_ (menatetrenone) suppresses bone mineral density (BMD) loss in the irradiated region after radiotherapy (RT) in uterine cancer patients.

**Materials and Methods:**

Our study included 34 patients who underwent whole pelvic irradiation for uterine cancer between 2001 and 2010. The patients were categorized in two groups: (1) Vitamin K_2_ (45 mg/day) administration group (group A) with 18 cases and (2) non-administered group (group B) with 16 cases. The duration of vitamin K_2_ administration was 1 year or longer. BMD was measured before and immediately, 3 months, 6 months, 1 year, 1 year or more after RT.

**Results:**

Regarding change rate in the BMD of L3-L4 which was outside the irradiated field, no significant changes were observed in BMD after radiation in either groups compared to BMD before radiotherapy. Regarding change rate in BMD of L5-S1 which was inside the irradiated field, BMD reduced significantly at 6 months after radiotherapy compared to BMD before the start of radiotherapy in Group B (*P* = 0.0234). However, no significant change was seen in group A. Grade 2 and 3 insufficiency fractures appeared in both groups, one in each. Regarding outside the irradiation field, one patient developed compression fracture in L2 in group B, none occurred in group A_._

**Conclusion:**

We suggest that vitamin K_2_ could suppress the decrease in BMD due to whole pelvic radiotherapy. Further studies are needed in the future to improve quality of life such as the prevention of insufficiency fractures.

## Introduction

Insufficiency fractures (IFs) of the lumbar spine and pelvic bone are one of the late adverse events after radiotherapy (RT) in gynecologic cancer patients [1–15]. Although there are differences in the irradiation method, the observation period and the modality used for diagnosis, the incidence of pelvic fractures including Ifs is 4.4–63% of patients if asymptomatic cases are included [1–15]. Several reports have evaluated considerable risk factors for fracture, such as advanced age [1, 4, 7, 9, 12, 13], low body weight or low body mass index (BMI)[1, 4, 13], many deliveries [1, 8], low bone mineral density (BMD) [12, 14, 15], postmenopausal status [3, 5, 7], radiation dose [9, 13], radiation method [13], brachytherapy [8], low CT densities [5, 11], rheumatoid arthritis [8]. Okonogi et al. reported that a decrease in BMD in the irradiated region after RT was observed within 1 year [6]. In addition to being painful, IFs can sometimes reduce a patient's quality of life.

Osteoporosis is a disease in which bone strength decreases due to decreased BMD and deterioration of bone quality [58]. We think that osteoporosis patients in RT for uterine cancer can be roughly divided into the following two patterns. ①Patients already osteoporosis before RT. ②Patients who develop osteoporosis due to RT. ② is further classified into (1) postmenopausal patients and (2) patients who underwent menopause due to RT. Here, we investigated whether BMD loss due to RT could be prevented by vitamin K_2_ and, as a result, whether IFs could be prevented. Although vitamin K_2_ is not covered by insurance as a prophylactic administration of osteoporosis, we hope that this will suppress BMD loss by RT.

Vitamin K is known to activate blood coagulation factors through it’s posttranslational modification of protein modules [16, 17]. In 1984, it was reported in the Lancet by Hart et al. that vitamin K_1_ levels were significantly lower in patients with osteoporosis who had vertebral compression fractures or femoral neck fractures, and the association between vitamin K and osteoporosis was also recognized internationally [18, 19]. In Japan, Kaneki et al. reported that the serum concentration of menaquinon-7, a form of vitamin K_2_, was significantly lower in elderly women with vertebral compression fracture than in those without fracture. These results suggest the possibility that deficiency of vitamin, particularly that of menaquinone-7, is one of the risk factor of developing osteoporosis [20]. Recently, it has been demonstrated that vitamin K may a role in the maintenance and improvement of vertebral bone and pelvic bone mineral density (BMD) and in the prevention of fractures in postmenopausal women with osteoporosis [29–39]. Furthermore, menatetrenone, one of the forms of vitamin K_2_, has been shown to prevent bone loss induced by ovariectomy and prednisolone treatment in rats [24–26]. In addition, vitamin K_2_ inhibits bone resorption in vitro [27, 28].

Vitamin K is a fat-soluble vitamin involved in the carboxylation and activation of several vitamin K-dependent proteins. It occurs in two different forms, vitamin K_1_ and vitamin K_2_, which differ in the length and degree of saturation of the side chain. Vitamin K_1_ is predominantly found in green vegetables, whereas vitamin K_2_ (menaquinones) are synthesized by bacteria in fermented food (natto) and meat. Vitamin K_2_ have a longer half-time and higher bioactivity than vitamin K_1_ [17, 21]. As a result, vitamin K_2_ is more effective in carboxylating extrahepatic vitamin K-dependent proteins than vitamin K_1_ [22]. Vitamin K_2_ (menatetrenone) acts directly on osteoblasts and generates the γ-carboxyglutamic acid residue of osteocalcin, a bone matrix protein. Menatetrenone prevents apoptosis of osteoblasts, improves their function, and upregulates bone turn over markers, hence providing an osteoprotective effect. That is, menatetrenone promotes bone formation, and improves bone turnover. At the same time, menatetrenone suppresses bone resorption and improves the imbalance of bone metabolism in the patient with osteoporosis to maintain the bone mineral density. In particular, it has been demonstrated that the side chain of menatetrenone alone has a bone resorption inhibitory effect [23]. Vitamin K regulates osteoblastogenesis and osteoclastogenesis through the nuclear factor κB (NF-κB) signal transduction pathway. Furthermore, vitamin K_2_ reduces osteoclastic activity via different strategies and exerts an anabolic effect on the bone [34].

Herein, we investigated the inhibitory effect of menatetrenone on the bone mineral density loss for uterine cancer patients receiving radiotherapy.

## Materials and methods

### Patients

This present study was approved by the Institutional Review Board (IRB) of Hiroshima University (Registration number: E-1836). Thirty-four patients who underwent whole pelvic radiotherapy for uterine cancer at Hiroshima University Hospital from 2001 to 2010 were evaluated retrospectively. Thirty-two patients had cervical cancer, and 2 patients had endometrial cancer. A total of 24 patients received radical RT, and 10 patients received postoperative RT. Patients characteristics are shown in Table 1.

**Table 1:** Patient characteristics

	Group A (vitamin K_2_)	Group B (non-vitamin K_2_)	
Age (Mean)	30–81 (65)	34–86 (65)	NS
Menopause before RT	15 (83%)	12 (75%)	NS
Uterine cervical cancer:			
Radical RT			
FIGO stage			
I	1	0	
II	2	4	
III	7	6	NS
IVA	2	1	
IVB	1	0	
Postoperative RT			
FIGO stage			
I	3	2	
II	1	2	NS
Histology			
Squamous	17	14	
Adeno	0	1	
Uterine endometrial cancer			
Postoperative RT			
FIGO stage			
I	0	1	
III	1	0	NS
Histology			
Adeno	1	1	
L3-4 BMD (g/m2)	0.945 ± 0.161	0.905 ± 0.084	NS
L4-S1 BMD (g/m2)	0.720 ± 0.221	0.663 ± 0.122	NS

Patients were divided into the vitamin K_2_ administration group (group A) with 18 patients and the non-administered group (group B) with 16 patients. In both groups, mean age was 65 years old, menopausal patients before treatment (radical RT or surgery) were 83% and 75%, 44% were over 70 years old, respectively, with no significant difference between the two groups. None of the patients were receiving some treatment for osteoporosis in this study.

### How to administer vitamin K_2_

Oral administration of vitamin K_2_ for the purpose of preventing RT-induced osteoporosis is an off-label use in Japan. However, patients undergoing chemoradiotherapy for uterine cancer have a decrease in appetite and are prone to poor dietary intake and vitamin K deficiency due to side effects of chemoradiotherapy. Therefore, we think administering vitamin K to treat vitamin K deficiency may be justified.

Based on Orimo’s study [56], we decided on the vitamin K_2_ dose 45 mg/day. The treatment period in the above study was 6 months, with possible cases receiving treatment up to 9 months. The 1998 study by Orimo et al. also had a 6-month treatment period [57]. The treatment period in Shiraki et al.’s study was 2 years [38]. Based on these studies, we set a treatment period of 1 year to allow for minimum outpatient follow-up and evaluation. And proposed treatment for more than 1 year for patients who had no adverse events and were able to attend the outpatient clinic.

In group A, vitamin K_2_ administration was initiated at the start of radiotherapy, and with 15 mg tablet orally administered three times daily after each meals (45 mg/day). Because absorption of vitamin K_2_ is reduced on an empty stomach in patients [40], vitamin K_2_ was instructed to strictly adhere to the postprandial intake under the guidance of a pharmacist. Since vitamin K weakens the effects of warfarin, vitamin K_2_ was contraindicated in patients taking warfarin.

As part of outpatient consultation, we explained and recommended oral vitamin K_2_ to patients before RT to prevent osteoporosis caused by RT from 2001 to 2010, regardless of whether they has osteoporosis or not. In other words, we administered vitamin K_2_ to the patients who requested it without knowing whether the patient had been diagnosed with osteoporosis. The BMD of both groups is shown in Table 1. Regarding BMD, there was no statistically significant difference between the two groups at L3-4 outside the irradiation field and L5-S1 inside the irradiation field.

We explained to the patient as follows. “There are many reports that insufficient fracture due to RT. RT is thought to reduce BMD within the irradiation field. Decrease in BMD is thought to be one of the causes of insufficiency fractures. This is unpublished data, when I was working at other institution, I conducted a study in which patients treated with RT were assigned to a group taking with or without vitamin K_2_, and found that the group taking with vitamin K_2_ tended to suppress BMD loss compared to the group not taking with vitamin K_2_. I presented the results at the Japanese Society for Cancer Therapy [41]. We consider that IFs is more likely to occur when BMD lose by RT. Although vitamin K_2_ is not covered by insurance as a prophylactic administration of osteoporosis, we hope that this will suppress BMD loss by RT. Would you like to take vitamin K_2_ orally to prevent osteoporosis caused by RT?” After this explanation, patients who answered that they would take vitamin K_2_ orally provided verbal consent and recorded it in their medical records. In this study, medical record information was collected retrospectively, and based on the records, we established groups that were taking vitamin K_2_ and groups that were not taking vitamin K_2_. Patients during that period were given the opportunity to opt out of participating in the study. Thirty of the 34 cases (88%) were consecutive cases during the first 4 years (2001 to 2004), whereas the subsequent 4 cases (12%) were cases during nonconsecutive periods (2 cases in 2007, 2 cases in 2010).

In group A, one patient developed stomatitis with possible side effects of vitamin K_2_ 6 months after the start of oral administration and thereafter, discontinued it. No other minor or serious adverse events, such as gastrointestinal symptoms and liver dysfunction was recorded.

### Healthy dietary vitamin K oral intake and exercise habits

In our study, we did not specifically recommend or limit healthy dietary vitamin K oral intake for both groups. And we did not specifically recommend or limit exercise habits or sunbathing which are necessary for vitamin D production.

### Radiotherapy treatment policies

The radiotherapy treatment policies for cervical caner in our hospital has previously been described [42]. The external beam RT (EBRT) for uterine cancer is the same as for cervical cancer. EBRT was given to the whole pelvis using the four-field box technique with 18 MV linear accelerator unit. The daily fraction size was 1.8–2 Gy, 5 fractions weekly. The superior border of the anteroposterior-posteroanterior fields were the superior edge of lumbar vertebra 5 (L5), inferior border was the obturator foramen, and laterally to 1.5–2 cm outside of the true pelvis. The anterior border of the lateral fields was over the anterior edge of the pubic symphysis and the posterior border was the anterior surface of the sacrum. As for the patients who underwent radical radiotherapy, central shield was used after 30–40 Gy with external whole pelvic irradiation, and the total dose was 50 Gy. High-dose rate intracavitary brachytherapy was given with ^192^Ir micro-Selectron for 24 patients [44]. The dose at point A was 6 Gy per fraction, 1 or 2 fractions per week and the number of fractions was 3 or 4. Total treatment time was 6–7 weeks.

### Measurement of BMD

BMD was assessed via dual-energy x-ray absorptiometry (DQR4500A). We measured BMD of the third lumber spine to the first sacral spine. We considered the third and fourth lumbar spine to be outside the irradiation field, and the fifth lumber spine and the first sacral spine to be within the irradiated field. We calculated the average BMD of the third and fourth lumbar spine and the average BMD of the fifth and first sacral spine. BMDs were measured before RT and 1, 3, 6 months, and more than 1 year after RT.

BMD measurements by the DXA method can be calculated higher than actual values in patients with original vertebral compression fractures. BMD varies individually for each patient. Even if each patient has degenerative spondylosis, compression fractures, or calcification due to arteriosclerosis before RT, we think that these effects on BMD are not so significant because we observe change rate of BMD before RT and BMD over time. Therefore, we think it is better to evaluate change rate of BMD in “%” over time, rather than evaluating the actual measured values of BMD in “g/cm^2^” over time, as many researchers have stated in their papers [31, 38, 39, 41, 56]. In this study, there was one patient in group A who had a previous mild fifth lumbar vertebra compression fracture which within RT field before RT. This lumbar vertebra was not excluded from the BMD measurement for the above reasons. On the other hand, this patient's L5 BMD decreased slightly over time. We do not believe that age correction is important [55].

### Observation period

In this study, we limited the observation of IFs to one year because the administration period of vitamin K_2_ was approximately one year.

### Statistical analyses

Statistical analyses were performed using JMP Pro 14 (SAS Institute Inc., Cary, NC, USA). Wilcoxon signed-rank test adjusted by Bonferroni correction was used to compare the indices from the same group at different timings.

For all analyses, a *p* value < 0.05 was considered to be statistically significant.

## Results

### Changes in BMD of L3-4 (nonirradiated regions)

**Fig. 1:**
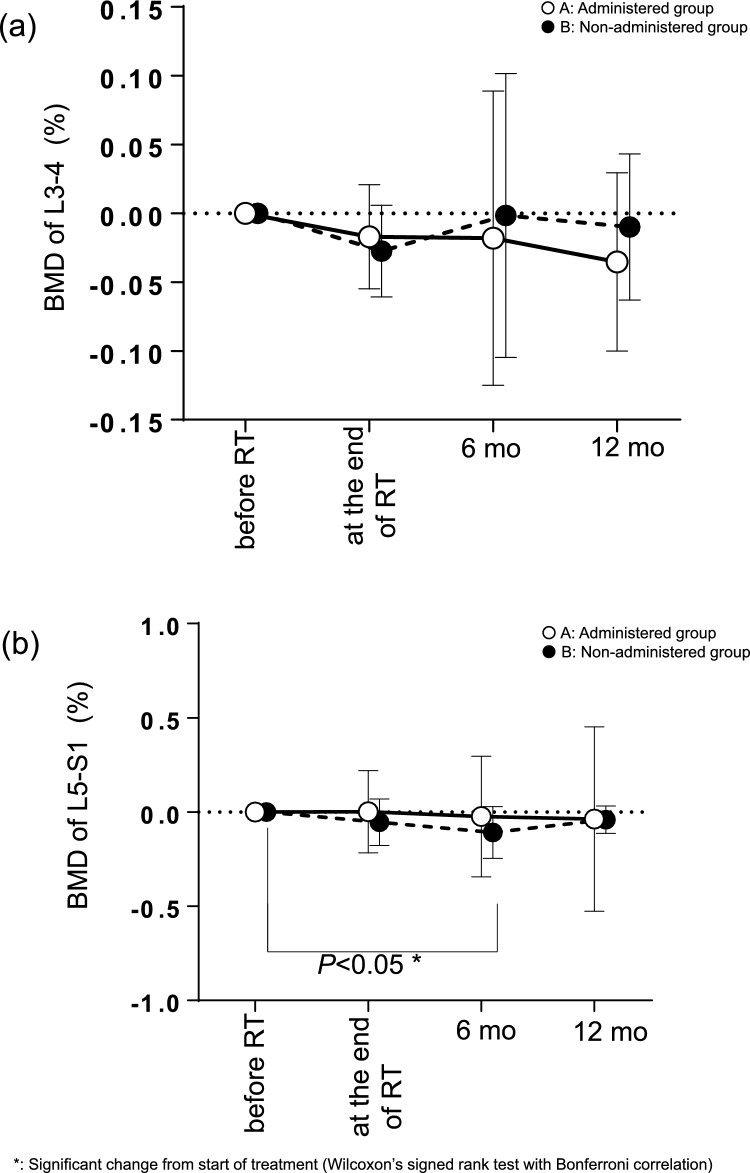
Change in BMD of (**a**) L3-4 nonirradiated regions and (**b**) L4-S1 irradiated regions The solid line shows the value of vitamin K2 (menatetrenone) administered group (n = 18), and the dashed line shows the value of the menatetrenone non-administerd group (n = 16)

Regarding change rate in the BMD of L3-L4 which was outside the irradiated regions based on the BMD value before RT, no significant changes were observed in BMD after RT in either groups (A and B) compared to BMD before the start of RT (Fig. 1a).

### Changes in BMD of L5-S1 (irradiated regions)

Regarding change rate in the BMD of L5-S1 which was inside the irradiated regions, the BMD reduced significantly at 6 months after RT compared to the BMD before the start of RT in group B (*P* = 0.0234). However, no significant change was seen in group A (Fig. 1b).

### Insufficiency fractures during the observation period after RT

**Fig. 2:**
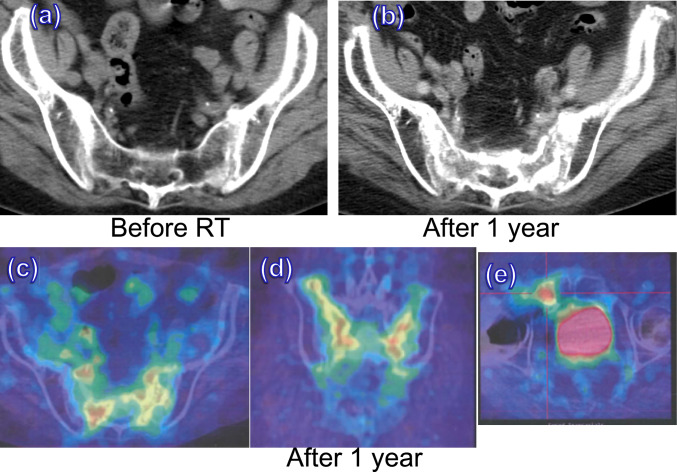
An 81-year-old woman who received RT alone for stage IIB cervical cancer with taking menatetrenone developed pain in lower back and buttocks without any particular incentive at 1 year after RT. She experienced difficulty walking, and was hospitalizes for detailed examination and treatment. (**a**) before RT (**b**) 1 year after RT: IF of sacroiliac joints (**c**) PET-CT 1 year after RT: uptake of bilateral sacroiloac joints increased; axial image (**d**) coronal image (**e**) IF of right pubic bone: uptake of right pubic bone increased

**Fig. 3:**
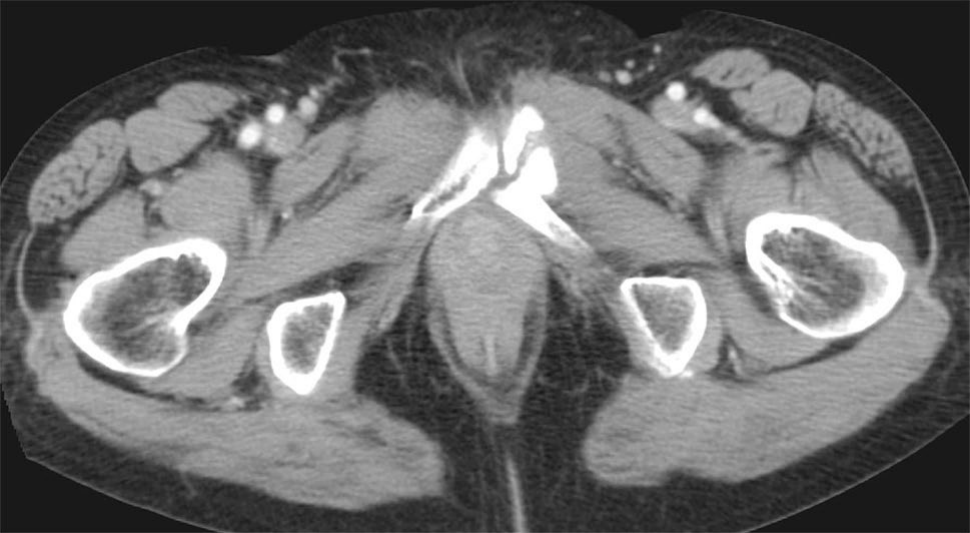
A 69-year-old woman who received chemoradiotherapy for stage IIB cervical cancer without taking menatetrenone developed pain in the left lower back and hip when waking up in the morning at 6 months after RT. She experienced difficulty walking due to hip pain, and received conservative treatment as outpatient

During the observation period within 1 year after RT, there were two new cases each in groups A and B (four cases in total) of insufficiency fractures (IFs) within the radiation field. The IFs of these four patients did not affect the BMD measurements in this study for the following reasons. The BMD was not measured at the site of the IFs because it was outside the imaging range, such as the pubic bone, or the IFs occurred after the measurement had been completed. Grade 2 and 3 insufficiency fractures appeared in both groups, one in each according to RTOG/EORTC late radiation morbidity [43]. Age of these 4 patients with IFs was 81 and 73 years old in group A, 63 and 69 years old in group B. IFs were observed at L5 in one patient in group A. Pubic bone fracture and osteosclerotic change of the sacro-iliac joint and L5 for one patient in group A (Fig. 2). IFs were observed at L5 for one patient in group B. One patient who developed a pubic bone fracture (Fig. 3) also developed a compression fracture in L2 which was outside the irradiation field at the same time in group B. Two patients with pubic bone fractures (one in each group) had temporary difficulty walking due to pain. On the other hands, regarding the outside the irradiation field, as mentioned above there was one case of a compression fracture of L2 in group B, but no IFs in group A without significant difference (*P* = 0.2817). Patients who developed IFs after radiotherapy in both groups A and B had lower mean BMD of L3, 4, 5, and S1 before RT compared with those who did not develop IFs without significant differences (Table 2a, b). Mean BMD before RT for patients who subsequently develop IFs were 0.780 g/m^2^ for L3-4 (out of field), and 0.481 g/m^2^ for L5-S1 (in field). On the other hand, mean BMD before RT for patients who do not subsequently develop IFs were 0.940 g/m^2^ for L3-4 (out of field), and 0.716 g/m^2^ for L5-S1 (in field).

**Table 2:** a BMD before RT for patients who subsequently develop IFs. b BMD before RT for patients who do not subsequently develop IFs

Group	Case No	Age	BMD (g/m^2^)		Site of subsequent IFs
			L3-4	L5-S1 (in field)	
(a)					
A (vitamin K2)	1	81	0.926	0.539	Pubic bone, sacroiliac joint
	2	73	0.648	0.426	L5
Mean		77	0.787	0.426	
B (non-vit K2)	3	63	0.714	0.502	L5
	4	69	0.816	0.456	Pubic bone, L2
Mean		66	0.765	0.479	
Total (mean)		72	0.780	0.481	

Group		Age	BMD (g/m^2^)		
			L3-4	L5-S1 (in field)	
(b)					
A (vitamin K2)	Mean	64	0.969	0.754	
B (non-vit K2)	Mean	65	0.912	0.678	
Total (mean)			0.940	0.716	

## Discussion

To our knowledge, this is the first paper to investigate the possible role of vitamin K_2_ in the prevention of osteoporosis caused by radiation therapy. Vitamin K_2_ has been shown to prevent bone loss induced by ovariectomy and prednisolone treatment in rats [24–26]. In addition, vitamin K_2_ has regulating effect on bone formation by gamma glutamyl carboxylation of osteocalcin. Furthermore, vitamin K2 enhanced not only the accumulation of Gla osteocalcin, but also osteocalcin production induced by 1,25(OH)2D3 in human osteoblasts in culture [27]. Vitamin K_2_ inhibits bone resorption partly through inhibition of prostaglandin E_2_ synthesis in vitro [28, 29]. BMD increased from baseline in patients received vitamin K_2_ [37].

In this study, oral administration of vitamin K_2_ suppressed bone mineral density loss in the irradiated field; however, it could not prevent fracture. Some reports suggest that vitamin K_2_ alone or in combination therapy improves BMD loss [37, 38].

### Vitamin K_2_ indications and side effects

Vitamin K_2_ can be prescribed to anyone, whether or not they are menopausal. Vitamin K_2_ is cheaper than bisphosphonate, and easy to use without serious side effects. As for economic analysis, vitamin K shows potential to be the most cost-effective treatment for preventing fractures compared alendronate, residronate, and strontium ranelate [33]. Furthermore, there are few serious side effects of this drug [37]. Stevenson reported that there are no adverse events as associated with vitamin K use [33].

In this study, only one patient developed stomatitis with possible side effects of vitamin K_2_ 6 months after the start of oral administration. And no other minor or serious adverse events occurred such as gastrointestinal symptoms and liver dysfunction. On the other hand, estrogen and selective estrogen receptor modulators (SERMs) are not targeted for premenopausal patients, but for postmenopausal osteoporosis patient. Radiotherapy eliminates ovarian function in premenopausal patients and leads to menopause. So, for premenopausal patients, the SERMs (raloxifen hydrochloride) would be indicated if the patient has osteoporosis after RT.

Due to the insurance system or it’s antiangiogenic effect [5, 48] that increases bone mass but deteriorates bone quality, it is difficult to administer bisphosphonates for the prevention of osteoporosis due to RT. We think that compared to other drugs, vitamin K_2_ has fewer side effects and is easier to administer orally.

### Vitamin K and dietary intake in relation to BMD

Kim MS suggests that an increase in dietary vitamin K intakes for maintaining BMD [45]. Vitamin K_2_ (Menaquinone-7) was effective for improving osteocalcin γ-carboxylation even at a low-dose daily intake [46]. In addition, the effect of low dose vitamin K_2_ (MK-4) supplementation on bioindices in postmenopausal Japanese women have been reported [47]. In our study, we did not specifically recommend or limit dietary vitamin K intake. Therefore, the results of our study do not take into account the effects of dietary vitamin K_2_ intake in patients’ daily meal.

### Why IFs occurred even in the group receiving vitamin K_2_ ?

In our study, BMD could be maintained by vitamin K_2_; however IFs could not be prevented and therefore, occured similar to group B (non-administerd group). That is, the vitamin K_2_ group had the same two cases of compression fractures as the group not receiving vitamin K_2_.

In the vitamin K_2_-treated group (group A) and the non-administered group (group B), patients who developed IFs after RT had a lower BMD before RT than patients who did not develop IFs. In our study, due to the small number of cases, no statistical analysis was performed. However, to confirm this observation, a study with a larger sample size is needed. We consider that for patients with low BMD before RT, the effect of vitamin K_2_ may have been insufficient even if BMD loss is suppressed after RT.

In addition to such low BMD before irradiation, we think that the causes of IFs are various. Ewing first reported the reaction of bone under RT as radiation osteitis [52]. Hasue M. et al. mentioned that broadly divided, the causes of IFs are as follows [53]. ①disorder of bone cell components ②vascular disorder ③ other. ①Osteoblast disappeared and osteoclast remain after RT in animal experiment. As osteoclasts are more resistant to radiation than osteoblasts, the balance between bone formation and resorption is disrupted. ②Bone atrophy occurs due to obliterative endovasculitis, venous congestion, leading to pathological fractures. Radiation causes bone matrix damage, increases bone marrow fat degeneration, and reduces bone vascularization [54], which contributes to the direct effects of radiotherapy on bone function and strength. ③RT causes bone demineralization due to changes in ionic binding of proteins and changes in cell membrane permeability. Individual differences: osteoporosis before RT, changes in the vascular system before RT, and endocrine system status.

Vitamin K_2_ has been reported to inhibit bone resorption through the inhibition of prostaglandin synthesis and osteoclast formation. That is, vitamin K_2_ suppresses osteoclast formation and promotes osteoblast activation and promotes improvement of bone metabolism.

However, we think that vitamin K_2_ cannot prevent deterioration of bone quality due to vascular damage caused by RT, so vitamin K_2_ may not have completely prevented IFs. In other words, among the causes of IFs caused by RT listed above, vitamin K_2_ will be able to suppresses ①, but will not be able to suppress ② and ③. In addition, there are various causes as mentioned next in “**Vitamin K and the prevention of non-irradiated bone fracture”**.

### Vitamin K and the prevention of non-irradiated bone fracture

In the outside the irradiation field, there were no IFs in group A. On the other hand, in group B, one patient who developed a pubic bone fracture at 6 months after RT also developed a compression fracture in L2 which was outside the irradiation field at the same time without significant difference (*P* = 0.2817). Significant differences may emerge as the number of cases increases. We consider that this may be due to the effect of vitamin K_2_.

The systemic review and meta-analysis of randomized controlled trials suggest that supplemention with vitamin K (vitamin K_1_ vitamin K_2_) reduces bone loss. Cockayne et al. noted that menaquinone-4 reduces bone loss and there is strong effect on incident fractures among Japanese patients [37]. Ushiroyama reports that continuous combined therapy with vitamin K_2_ and vitamin D_3_ may be useful for increasing vertebral bone mineral density in postmenopausal women compared vitamin K_2_ or vitamin D_3_ alone [31].

A meta-analysis of 19 randomized controlled trials showed a significant improvement in vertebral BMD amongst vitamin K_2_ receivers. Furthermore, a sensitivity analysis carried out by rejecting studied with heterogeneity demonstrated a significant difference in the incidence of fractures favoring vitamin K_2_ [34].

Vitamin K_2_ is said to be effective in preventing fractures independent of an increase in bone density. Kashima et al. reported that after administration of vitamin K_2_, in morphorogy images, marked improvement was noted in the distribution and density of the skeletal pattern of the femur and the third lumber vertebra in the patient with osteoporosis [30].

Shiraki et al. reported that vitamin K_2_ (menatetrenone) treatment for osteoporotic patients effectively prevented new fractures, although there was no increase in lumber BMD (LBMD) in the vitamin K_2_-treated group. However, fracture incidence in the vitamin K_2_-treated group was significantly lower than that in the control group [38].

Regarding the prediction and evaluation of the therapeutic effects with vitamin K_2_, Shiraki mentioned that vitaminK_2_ only has the effect of maintaining lumbar vertebrae BMD, but its fracture prevention effect is equivalent to that of other drugs [47]. He stated that the effects of vitamin K_2_ vary greatly from person to person, and one of what determines individual differences is the apolipoprotein E phenotype [49]. In patients with apolipoprotein E3-/- phenotype group, not only the administration of vitamin K_2_ was ineffective, but BMD tended to decrease to the extent that it was considered a fast loser [49]. And Shiraki mentioned that a typical biological effect of vitamin K_2_ is promotion of Gla conversion of vitamin K-dependent Glu-containing proteins. The high Glu-OC (under-carboxylated osteocalcin) group, in which Glu-OC did not decrease even after vitamin K_2_ was administered, had no significant effect on BMD compared to the low Glu-OC group [50].

The therapeutic effect of vitamin K_2_ must be evaluated comprehensively, including changes in internal skeletal structure, rather than being evaluated solely by increases or decreases in BMD.

Furthermore, it is conceivable that there may be patients in whom improvement in BMD and bone strength do not correlate. Kashima et al. mentioned that vitamin K_2_ administration improves skeletal structure and contributes to reducing the risk of fractures using morphology processing image (BMD increases). On the other hand, in some cases, increases or decreases in BMD did not necessarily correlate with changes in internal skeletal structure. (BMD increased but skeletal structure deteriorated; BMD decreased but skeletal structure remained unchanged). They mentioned that the therapeutic effect of vitamin K_2_ must be evaluated comprehensively, including changes in internal skeletal structure, rather than being evaluated solely by increases or decreases in BMD [51].

As mentioned above, some studies reports that the combination of vitamin D_3_ and calcium with vitamin K_2_ increases bone density and strength. In the future, the combined use of these drugs should be considered for the prevention of IF. Diet and vitamin D_3_ are also important [31]. There are reports that age, menopause, long-term use of corticosteroid, osteoporosis, low BMD, irradiation, and sacral D_50_ [9] are risk factors for fractures [2, 4, 5, 10]. In our study, menopausal patients were 83% and 75% in group A and B, respectively. And 2 patients each who developed IFs in group A and group B, were also elderly. The IF incidence rate of patients treated with RT technique with anteroposterior fields is 2.6–4.3 times higher than that of IMRT [10]. Uezono and Ishikawa [5, 11] et al. reported that the low pretreatment CT densities of spinal and pelvic bones before RT was risk factor of IF. Kurrumeli et al. reported that one of the predisposing factors for developing PIF (pelvic IF) after radiotherapy seems to be the low BMD [15]. The BMD of the sacral bone significantly lower in the PIF group compared in the OTH (other patients) group. They noted that there was a significant difference of the mean BMD of the lumber vertebrae in the PIF group and OTH group. We think that for patients at risk of osteoporosis, irradiation methods that reduce bone volume, such as IMRT, may need to be devised.

In addition to vitamin K_2_, combined administration of such as vitamin D_3_, calcium, SERM (for postmenopausal osteoporosis patient) and careful observation and lifestyle guidance (dietary habits and exercise therapy) for cases in which IFs is likely to occur or low BMD before RT are considered important.

Our study has several limitations. First, this was a retrospective single-institution study and the small number of patients in both groups and small number of patients of IFs limited the statistical reliability. And the BMD observation period for many of the patients in this study was limited of one year, so long-term BMD changes could not be observed. Second, potential bias may exist because the study was not randomized and vitamin K_2_ intake was by patient’s own choice. Third, there may be a bias that IFs are inherently likely to occur regardless of irradiation because the average age is high in both groups. Forth, we did not investigate how patients actually behaved for oral vitamin K_2_ intake from diet, and exercise habits during the study period. Fifth, because the number of premenopausal patients was small, and premenopausal patients reaches menopause by RT, we did not separately examine the cases before and after menopause.

In conclusion, oral administration of vitamin K_2_ (menatetrenone) suppressed BMD loss due to RT in the irradiated field after RT. However, the bones after RT appeared fragile, and oral administration of vitamin K_2_ could not prevent fractures enough in the irradiation regions in this study.

Subsequent quality of life in patients with uterine cancer after RT is important. We believe that it is important to continue research to prevent reduce of BMD and IFs due to RT. Further investigation is required by increasing the number of cases and periods.

## Data Availability

We cannot share data openly for reasons of protecting the privacy of patients.
